# Supplementary material for: Synthesis and Properties of a Bay-Annulated-Indigo Tetramer Based on Low-Cost Spiro[Fluorene-9,9′-Xanthene] Core
Source: Molecules. 2019 Oct 8;24(19):3623. doi: 10.3390/molecules24193623 (PMC6804169; doi:10.3390/molecules24193623)
Supplement: Supplementary file 1 [file molecules-24-03623-s001.pdf]

Synthesis and Properties of a Bay-Annulated-Indigo Tetramer Based on Low-Cost Spiro[Fluorene-9,9′-Xanthene] Core

Bao-Yi Ren [1,*], Yan-Mei Tan [1], Chang-Liang Sun [1] and Yong-Gang Sheng [1,2]

[1] College of Applied Chemistry, Shenyang University of Chemical Technology, Shenyang 110142, China; m15124190633@163.com (Y.-M.T.); chemscl@126.com (C.-L.S.); gara@syuct.edu.cn (Y.-G.S.)

[2] Key Laboratory for Organic Electronics and Information Displays & Institute of Advanced Materials (IAM), Nanjing University of Posts and Telecommunications, 9 Wenyuan Road, Nanjing 210023, China

* Correspondence: renbaoyi@syuct.edu.cn; Tel.: +86-1388-988-1669

Contents:

1. Cost estimations 1

2. The characterization data of intermediates and target compounds 2

3. TGA curves 4

4. Summary of UV-vis, CV, and DFT calculations 4

References 5

1. Cost estimations

Cost estimations of 4Br-SFX and bromo-BAI were adapted from [1] and [2].

***Table S1**. Material quantities and costs for the synthesis of 4Br-SFX.*

Chemical name	Weight reagent (g/g)	Weight solvent (g/g)	Weight workup (g/g)	Price of Chemical ($/kg)	Material cost ($/g product)	Total cost ($/g)
2,7-dibromo-9*H*-fluoren-9-one	2.33			285	0.23	3.20
3-bromophenol	11.92			150	0.60	
$MeSO_3H$	3.98			60.5	0.08	
EtOH			50	5.8	0.22	
Ethyl acetate			40	3.4	0.20	
petroleum ether			240	2.9	0.54	
Silica gel			20	8.0	0.13	

***Table S2**. Materials quantities and cost for the synthesis of bromo-BAI.*

Chemical name	Weight reagent (g/g)	Weight solvent (g/g)	Weight workup (g/g)	Price of chemical ($/kg)	Material cost ($/g product)	Total cost ($/g)
Indigo	5.13			7.40	0.038	5.49
Acetylchloride	3.86			11.85	0.046	
acetic anhydride		5.90		5.17	0.03	
2-Thiopheneacetyl chloride	1.90			427.57	0.81	
2-(4-((2-ethylhexyl)oxy)phenyl)acetyl chloride	4.20			220.50	0.92	
NBS	1.68			37.28	0.06	
1,4-Dioxane		4.30		11.20	0.05	
Xylene		5.00		10.40	0.05	
Dichloromethane			500	2.14	1.07	
Silica gel			80	8.0	0.64	
Chloroform			500	3.56	1.78	

2. Characterization data of intermediates and target compounds

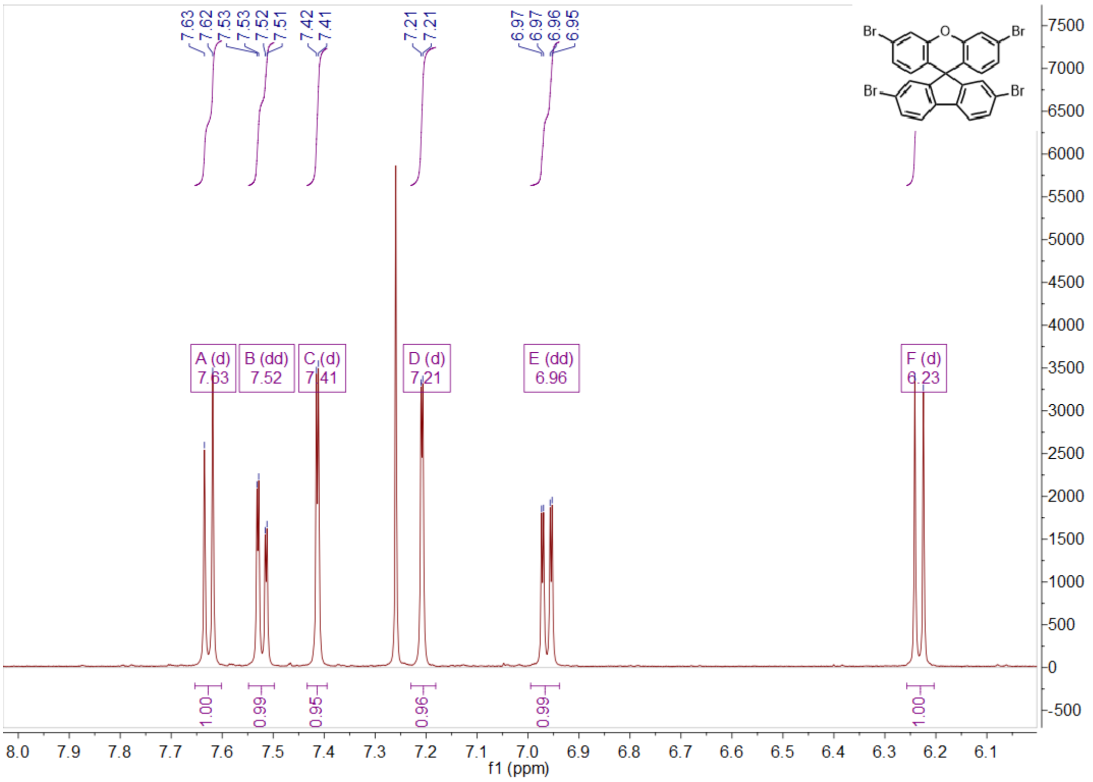

Figure S1. [1]*H NMR spectra of 4Br-SFX ($CDCl_3$, 298K).*

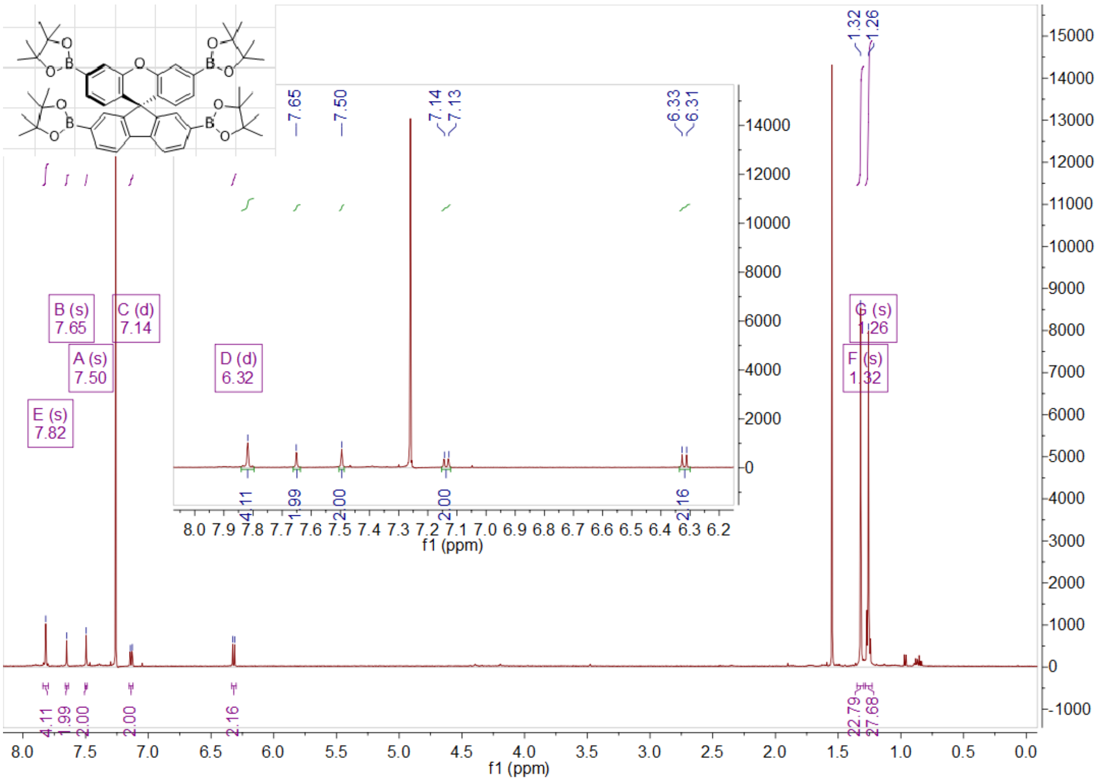

Figure S2. [1]*H NMR spectra of 4Bpin-SFX ($CDCl_3$, 298K).*

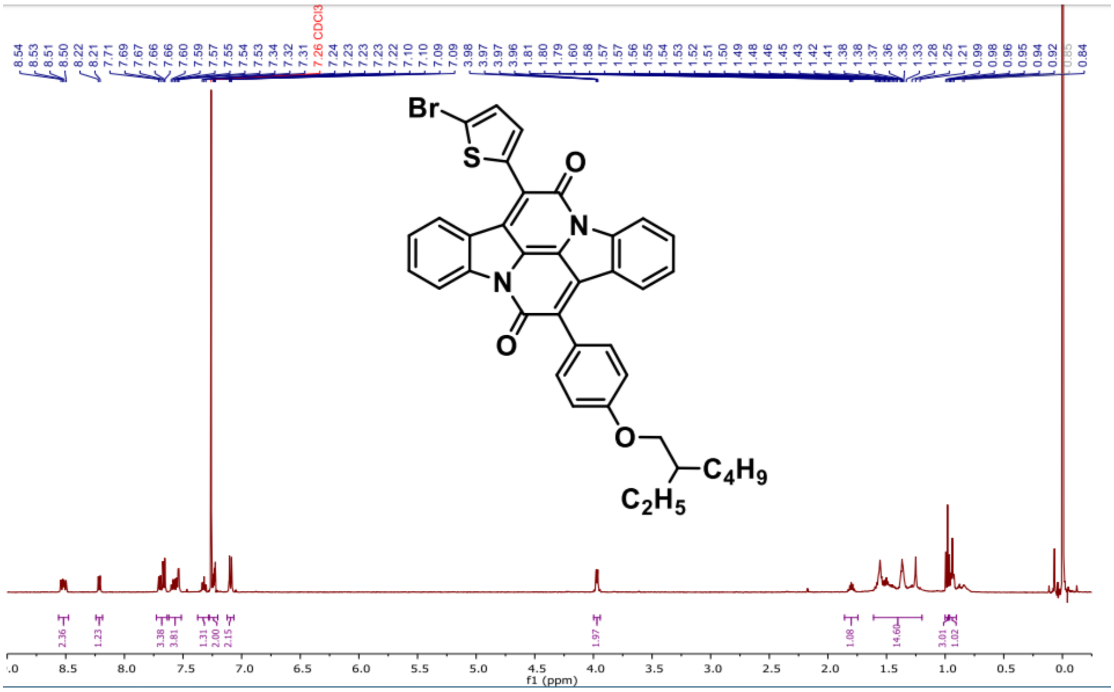

Figure S3. [1]*H NMR spectra of bromo-BAI ($CDCl_3$, 298K).*

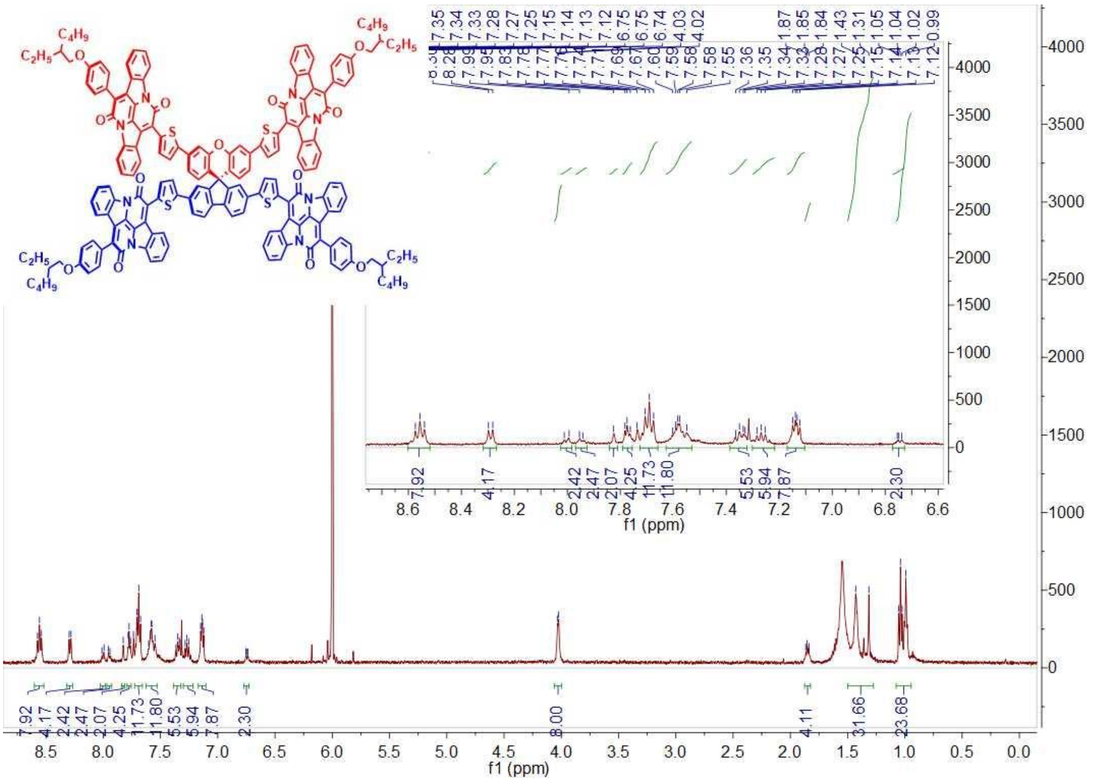

Figure S4. [1]*H NMR spectra of* ***4BAI-SFX*** *($C_2D_2Cl_4$, 358 K)*.

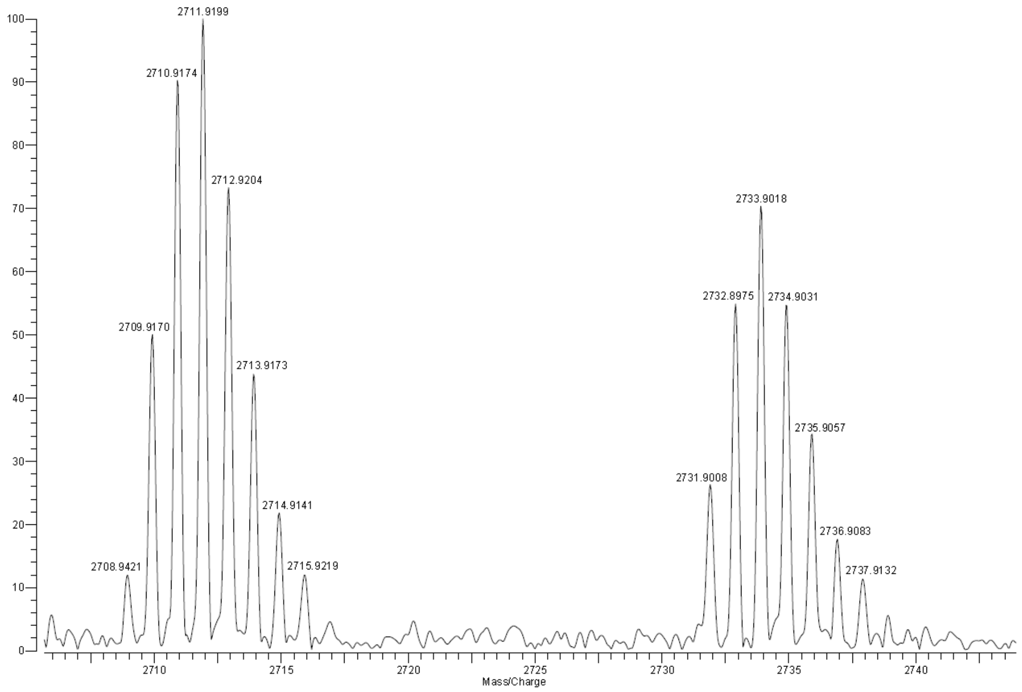

Figure S5. *MALDI-TOF spectrum for 4BAI-SFX.*

3. Thermogravimetric analysis (TGA) curves

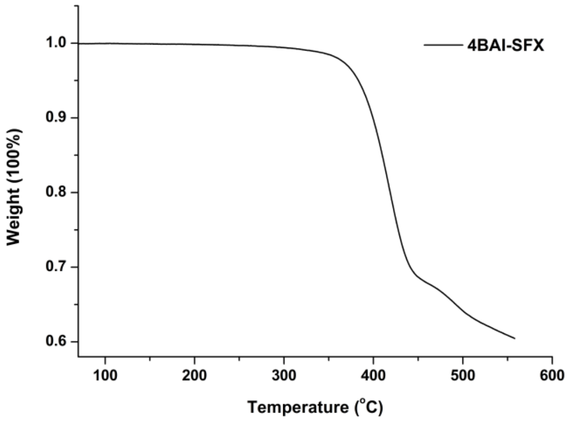

Figure S6. *Thermogravimetric analysis (TGA) curves of 4BAI-SFX in a nitrogen atmosphere.*

4. Summary of UV-vis, CV, and DFT calculations

Table S3. *Summarized results of photophysics, electrochemistry, and DFT calculations.*

Compd.	UV-Vis[a]				Cyclic Voltammetry[b]			Calculated FMOs		
	λ_{max} /nm	ε_{max} /M^{-1} cm^{-1}	λ_{onset} /nm	E_g^{opt} /eV	E_{HOMO} /eV	E_{LUMO} /eV	E_g^{Elec} /eV	E_{HOMO} /eV	E_{LUMO} /eV	E_g^{Calc} /eV
**4BAI-SFX**	596	145300	687	1.80	−5.34	−3.59	1.75	−4.93	−2.84	2.09

[a] Measured in dilute chloroform solutions, [b] samples were prepared in degassed $CHCl_3$ solution with tetrabutylammonium hexafluorophosphate (0.1 M) as the electrolyte at a scan rate of 100 mV s^{-1}.

References

[1] M. Maciejczyk, A. Ivaturi, N. Robertson, SFX as a low-cost ‘Spiro’hole-transport material for efficient perovskite solar cells, Journal of Materials Chemistry A, 4 (2016) 4855-4863.

[2] B. He, A.B. Pun, D. Zherebetskyy, Y. Liu, F. Liu, L.M. Klivansky, A.M. McGough, B.A. Zhang, K. Lo, T.P. Russell, New form of an old natural dye: bay-annulated indigo (BAI) as an excellent electron accepting unit for high performance organic semiconductors, Journal of the American Chemical Society, 136 (2014) 15093-15101.
